# Complete mitochondrial genome of *Linoclostis gonatias* (Lepidoptera: Xyloryctidae)

**DOI:** 10.1080/23802359.2020.1791746

**Published:** 2020-07-23

**Authors:** Tianjuan Su, Kai Jiang, Bo He, Fang Zhao, Gonghua Lin, Zuhao Huang

**Affiliations:** School of Life Sciences, Jinggangshan University, Ji’an, China

**Keywords:** Xyloryctidae, *Linoclostis gonatias*, mitogenome, phylogeny

## Abstract

The complete mitochondrial genome of *Linoclostis gonatias* (Lepidoptera: Xyloryctidae) is 15,528 bp in length, containing 13 protein-coding genes, 22 transfer RNAs, 2 ribosomal RNAs, and a putative control region. Except for *cox1* starts with CGA, all other PCGs use the typical ATN codons. Most of the PCGs end with the complete stop codon TAA, whereas *cox2* terminates with the incomplete stop codon T. The BI analysis was performed using a dataset matrix containing 13 PCGs concatenated from the mitogenomes of Gelechioidea species. The monophyly of Xyloryctidae was highly supported. In addition, Oecophoridae was inferred as the sister group of Xyloryctidae.

The Xyloryctidae was often considered to be a subfamily of Oecophoridae, however, Hodges (1999) raised it to family level. It is worldwide in distribution with more than 1200 species in 86 genera (Hodges, 1999). To date, only one complete mitochondrial genome (mitogenome) has been sequenced from members of this family. In this study, we present the complete mitogenome of *Linoclostis gonatias* (Lepidoptera: Xyloryctidae). It is reported as the major pest of *Camellia oleifera*, which is the most economically important woody oil tree in China.

The specimen was collected from Jiangxi Province, China (27.922227, 115.087795) in June 2019, and was preserved at Entomological Specimen Room of Jinggangshan University (accession number: 20190601S). Total genomic DNA was extracted from a single specimen and was sequenced by Illumina HiSeq2000, with a read length of 150 bp. The complete mitogenome was assembled by the GetOrganelle v1.6.4 program (Jin et al. 2018) and was annotated using the MITOS2 webserver (Bernt et al. 2013).

The complete mitogenome of *L. gonatias* is a circular molecule of 15,528 bp in length. It contains the typical set of 37 genes as in most insect mitogenomes (Cameron 2014), including 13 protein-coding genes (PCGs), 2 ribosomal RNAs (*rrnL* and *rrnS*), 22 transfer RNAs (tRNAs), and a non-coding control region. The J-strand encodes most of the genes (9 PCGs and 14 tRNAs), while the remaining genes (4 PCGs, 8 tRNAs, and 2 rRNAs) are located on the N-strand. The overall base composition is 39.3% A, 40.9% T, 12.2% C, and 7.5% G. The nucleotide composition of the *L. gonatias* mitogenome is biased toward A + T (80.2%). All of the PCGs use the standard start codon ATN, except for *cox1* which starts with CGA. Twelve PCGs use the common stop codon TAA, however, *cox2* terminates with the incomplete stop codon T. The *rrnL* is located between *trn-Leu1* and *trn-Val*, with the length of 1345 bp. The *rrnS* is located between *trn-Val* and the control region, with the length of 791 bp. The control region is 313 bp in length, and is located between *rrnS* and *trn-Ile*. Like the majority of other lepidopterans (Wang et al., 2016), it is characterized by its remarkably high A + T content of 95.2%.

Phylogenetic analysis was performed on concatenated nucleotide sequences of the 13 PCGs of nine Gelechioidea species, along with five outgroup species (Figure 1). The best-fit partitioning scheme and nucleotide substitution model were simultaneously confirmed with PartitionFinder 2.1.1 (Lanfear et al. 2016). Phylogenetic inference was performed using MrBayes 3.2.6 (Ronquist et al., 2012) through the online CIPRES Science gateway (Miller et al., 2010). Within the Gelechioidea, *L. gonatias* was grouped with *Opisina arenosella* as the sister group. Accordingly, the monophyly of Xyloryctidae was highly supported. In addition, Oecophoridae was inferred as the sister group of Xyloryctidae.

**Figure 1.**
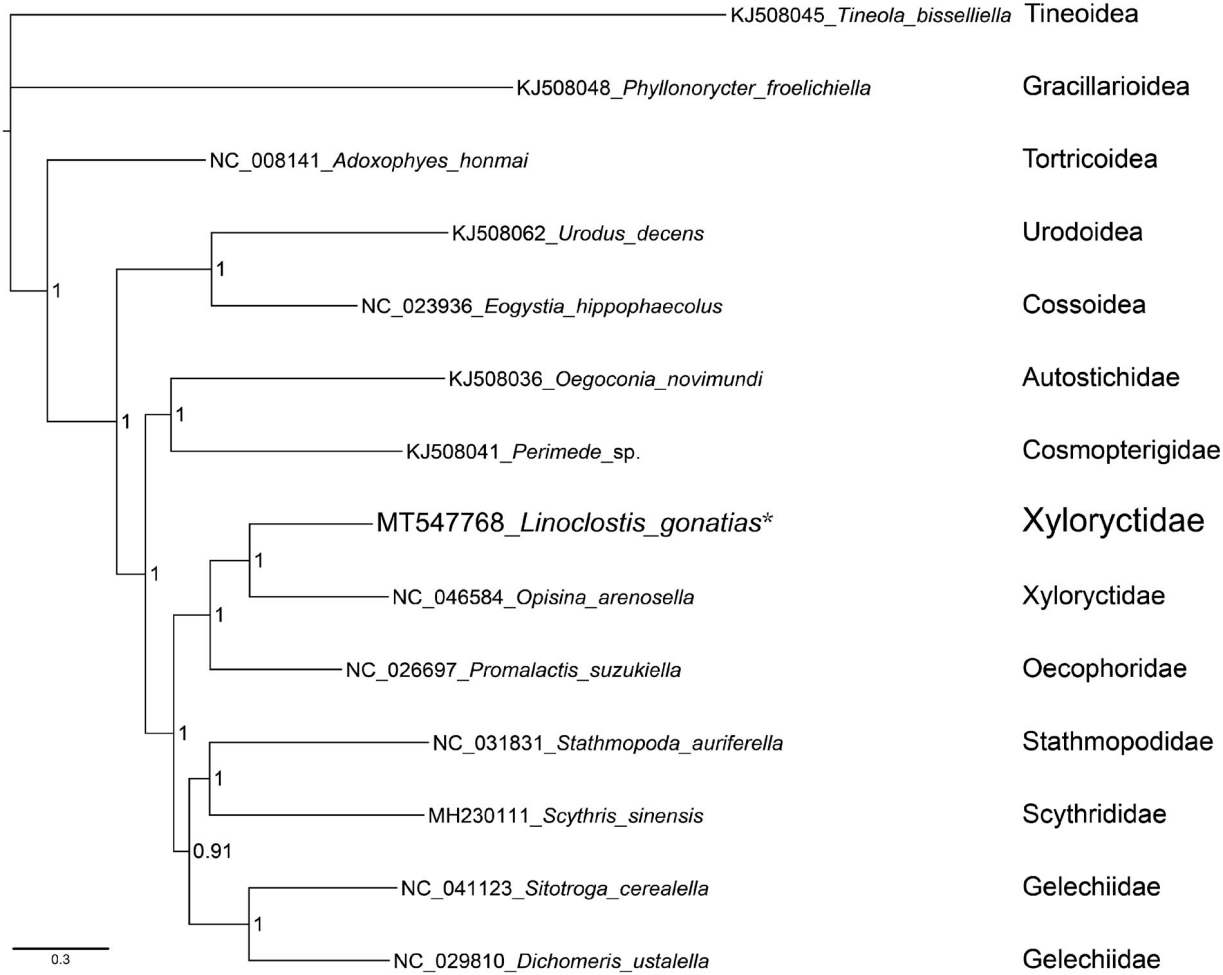
Phylogenetic relationship of Gelechioidea based on the BI analyses of the concatenated sequences of 13 PCGs. Numbers on branches are Bayesian posterior probabilities.

## Data Availability

The data that support the findings of this study are available in [GenBank] at [https://www.ncbi.nlm.nih.gov/nuccore/MT547768], reference number [MT547768].
